# Doxycycline Hydrochloride Regulates Cytoskeletal Rearrangement and Epithelial-To-Mesenchymal Transition in Malignant Rhabdoid Tumour of the Kidney

**DOI:** 10.1155/2022/2760744

**Published:** 2022-11-09

**Authors:** Chenghao Zhanghuang, Zhaoxia Zhang, Tao Mi, Jinkui Wang, Liming Jin, Jiayan Liu, Maoxian Li, Mujie Li, Xin Wu, Zhang Wang, Dawei He

**Affiliations:** ^1^Department of Urology, Chongqing Key Laboratory of Children Urogenital Development and Tissue Engineering, Chongqing Key Laboratory of Pediatrics, Ministry of Education Key Laboratory of Child Development and Disorders, National Clinical Research Center for Child Health and Disorders, China International Science and Technology Cooperation Base of Child Development and Critical Disorders, Children's Hospital of Chongqing Medical University, Chongqing Higher Institution Engineering Research Center of Children's Medical Big Data Intelligent Application, Chongqing, China; ^2^Department of Urology, Kunming Children's Hospital, Yunnan Provincial Key Research Laboratory of Pediatric Major Diseases, Kunming Medical University, Yunnan Province Clinical Research Center for Children's Health and Disease, Kunming, China

## Abstract

**Objective:**

As a highly malignant tumour, malignant rhabdoid tumours of the kidney (MRTK) are prone to metastasis and invasion, while tumour metastasis and invasion are inseparable from matrix metalloproteinases (MMPs) and epithelial-mesenchymal transformation (EMT). Moreover, the key to EMT is remodelling of the cytoskeleton. Therefore, our study is aimed at investigating whether doxycycline hydrochloride (DCH), an inhibitor of MMPs, could reverse EMT in MRTK to exert an antitumour effect by regulating MMPs and the cytoskeleton.

**Methods:**

The existence of EMT in MRTK cells was verified by bioinformatics analysis, immunofluorescence, and western blotting (WB). *In vitro*, the proliferation, migration, and invasion abilities of G401 cells were examined by Cell Counting Kit-8 (CCK-8), scratch, and Transwell assays, respectively. The effect of DCH on tumour growth in tumour-bearing mice was explored in *in vivo* experiments, and the expression of MMP2 and MMP9 and EMT correlation indexes was measured by immunofluorescence and WB, and the changes in cytoskeletal F-actin and *β*-tubulin were measured by fluorescence.

**Results:**

The altered extracellular matrix (ECM) composition, EMT, and high expression of MMP2 and MMP9 existed in MRTK. DCH inhibited the proliferation, migration, and invasion of G401 cells *in vitro. In vivo*, DCH inhibited tumour growth in mice, downregulated the expression of MMP2 and MMP9, and partially reversed EMT. Alternatively, DCH resulted in cytoskeletal rearrangements of G401 cells.

**Conclusions:**

DCH, as an MMP inhibitor, is used for the first time in MRTK research, showing good antitumour effects by reversing EMT and potentially providing new therapeutic measures for MRTK treatment.

## 1. Background

Malignant rhabdoid tumours (MRTs) are rare and highly aggressive malignant tumours that mainly occur in children under the age of 3 years and can occur in a variety of tissues and organs throughout the body, mainly in the kidney and central nervous system [1, 2]. Among them, MRTs occurring in the kidney, defined as malignant rhabdoid tumours of the kidney (MRTK), are the most common type of MRTs [3, 4]. MRTK accounts for approximately 0.9%-2% of renal tumours in children [5]. The majority of MRT patients receive an intensive multimodal treatment regimen with a combination of surgery, radiotherapy, and chemotherapy, which may provide a significant increase in survival benefits for MRT patients. Despite aggressive treatment, the long-term survival rate of MRT patients is only 15-50% [6] due to drug resistance and ease of distant metastasis [7], while the overall survival rate of MRTK patients is even lower than 25% [8]. Therefore, improving the survival rate of MRTK patients appears crucial.

Approximately 50% of children with MRTK have concomitant distant metastases at diagnosis, so MRTK cannot achieve complete remission by surgical resection, which is also an important reason for the high mortality rate of children with MRTK [9]. Most patients with malignancies also die from metastatic disease; therefore, the prevention of metastasis is the most critical factor in improving the cure rate of highly aggressive malignancies, including MRTK. However, due to its rare incidence, there are very few studies on MRTK, and no studies have explored the mechanism of MTRK metastasis. The mechanism of tumour metastasis is complex and involves many interactions between tumour cells and their microenvironment. Increasing evidence supports the idea that extracellular matrix (ECM) molecules show critical functional roles in the control of key cellular events. To migrate through the dense barrier of the ECM, cells need to degrade and remodel the ECM structure [10]. Matrix metalloproteinases (MMPs) mediate many changes in the microenvironment during tumour progression [11]. MMPs are a family of zinc-dependent ECM remodelling peptide endonucleases that were first described nearly half a century ago and can degrade almost all ECM components, especially collagen, in the ECM [12]. However, changes in the ECM composition can also induce EMT [13]. Epithelial-mesenchymal transformation (EMT) is considered necessary for tumourigenesis, enabling tumours to acquire invasive and metastatic capacity [14]. MMPs induce changes during EMT in malignancies and promote EMT through invasion and metastatic behaviour [15].

MMPs associated with cancer have been studied for over 40 years, and the idea that MMP-mediated degradation of ECM leads to cancer cell invasion and metastasis is a guiding principle for MMP research [16]. The migration and invasion of many malignancies are inseparable from the actions of MMPs, such as breast cancer, kidney cancer, and liver cancer [17–19], and high levels of MMPs are associated with the poor prognosis of many cancers [20]. Thus, between the 1990s and early 2000s, synthetic inhibitors of MMPs (MMPIs) were extensively studied in various cancer types [21, 22]. However, these studies failed due to a lack of efficacy and the incidence of severe side effects. Doxycycline hydrochloride (DCH) is a tetracyclic antibiotic produced by Streptomyces that has a significant inhibitory effect on the activity of MMPs; therefore, DCH is considered to have potential as an antitumour agent [23]. Studies have shown that DCH has a potent inhibitory effect on breast cancer tumour stem cells (BCSCs). Due to its low toxicity, combining DCH with chemotherapeutic drugs to eradicate BCSCs and large tumour cells is considered safe [24]. Zhang et al. confirmed that DCH inhibited breast cancer cell migration, invasion ability, and EMT [25]. Furthermore, in prostate cancer, DCH suppresses cell proliferation, invasion, and metastasis [26]. Therefore, DCH deserves extensive study as a promising antitumour drug; however, no research has focused on the role of DCH in MRTK.

As a broad-spectrum inhibitor of MMPs, although DCH can inhibit various MMPs, current research on DCH is still focused on MMP2 and MMP9. Studies have shown that the use of DCH reduces MMP2 and MMP9 activity in breast cancer and oral squamous cell carcinoma [27, 28]. Among all MMPs, MMP2 and MMP9 are also the most-studied MMPs in tumour EMT pathogenesis [29]. MMP2 and MMP9 can hydrolyse basement membrane components and regulate various aspects of tumour growth and metastasis [30]. Moreover, EMT plays a crucial role in the metastasis of malignant tumours [31]. Previous studies have found that EMT is associated with colorectal cancer and oral cancer metastasis [32–34]. The above evidence suggests that EMT, as well as MMP2 and MMP9, plays a key role in the pathogenesis and metastasis of malignancy. Do EMT, MMP2, and MMP9 also play a key role in the pathogenesis and metastasis of MRTK? Moreover, does DCH have an antitumour effect on MRTK? These questions are worth further discussion. Therefore, this study is the first to target MRTK, a rare tumour, in combination with the MMP inhibitor doxycycline, aiming to determine whether EMT, as well as the differential expression of MMP2 and MMP9, exists in MRTK. Second, we sought to explore whether DCH achieves antitumour growth effects by regulating MMP2 and MMP9 expression, thus reversing EMT, with a view to providing new potential therapeutic options for MRTK.

## 2. Materials and Methods

### 2.1. Bioinformatics Analysis

The mRNA expression data of MRTK were obtained through the R software R4.0.1 package (downloaded from the Therapeutically Applicable Research to Generate Effective Treatments (TARGET) database). The screening criteria were as follows: (1) data from primary tumour and normal samples and (2) data on mRNA expression available. Seventy samples were obtained, including 64 tumour samples and six adjacent standard samples. Kyoto Encyclopedia of Genes and Genomes (KEGG) pathway and Gene Ontology (GO) functional enrichment analyses were performed on the differentially-expressed mRNAs in R software using the cluster analysis software package. Enrichment analysis of biological processes (BPs), cell components (CCs), molecular functions (MFs), and KEGG pathways was conducted. Histograms were constructed using the ggplot2 package. An adjusted *p* value of <0.05 was used as the screening criterion.

### 2.2. Clinical Tissue Specimens and Cell Cultures

This study was approved by the Ethics Committee of Children's Hospital Affiliated with Chongqing Medical University and Kunming Children's Hospital. All patients and their parents signed informed consent forms before joining the study. A total of 6 patients with MRTK tumours and their adjacent tissues were collected. The histopathology of MRTK was determined by two chief pathologists. The MRTK cell line G401 was purchased from the Shanghai Cell Bank and cultured using DMEM supplemented with 10% foetal bovine serum (FBS) and penicillin–streptomycin at 37°C and 5% CO_2_.

### 2.3. Cell Viability Measurement by Cell Counting Kit-8 (CCK-8) Assay

The half-maximal (50%) inhibitory concentration (IC_50_) of DCH against G401 cells was first screened by CCK-8 assay, and 2500 G401 cells per well were plated into 96-well plates. G401 cells were then treated with DCH at different concentrations (0, 10, 20, 40, 60, 80, and 120 *μ*M) for 48 h to determine the IC_50_. Our subsequent cell experiments were performed at three concentrations: 1/2 IC_50_, IC_50_, and 2 IC_50_. Cell proliferation was also detected by CCK-8 assay. At 0, 24, 48, and 72 h after DCH intervention, 10 *μ*l of CCK-8 solution was added and incubated at 37°C for 2 h. Then, the absorbance was measured at 450 nm using a microplate reader, and the proliferation curve was plotted.

### 2.4. Cell Migration Ability Examination by a Scratch Experiment

G401 cells were plated into 6-well plates at 300,000 cells per well. After the cells adhered, a vertical wound was created with a 20 *μ*l gun head tip, washed using PBS, and treated with different concentrations of DCH. Cell scratches were photographed using a microscope at 0, 24, and 48 h, and cell migration was recorded.

### 2.5. Cell Invasion Ability Determination by Transwell Assay

After DCH intervention for 24 h, G401 cells were digested, centrifuged, and resuspended in serum-free medium. Then, 20,000 cells per well were seeded into the upper Transwell chamber prelaid with matrix glue, and 800 *μ*l of medium containing 10% FBS was added to the lower chamber. The cells were incubated in an incubator for 24 h, fixed with 4% paraformaldehyde, stained with crystal violet, washed in PBS, and dried. The upper cells were wiped off using cotton swabs and photographed under a microscope, and the cell numbers were counted using ImageJ.

### 2.6. Animal Experiments

All procedures in this study were approved by the Institutional Animal Care and Use Committee of Chongqing Medical University, and 4-week-old nude mice were selected for animal experiments. A total of 5^∗^10^6^ G401 cells were subcutaneously injected into the left axillas of nude mice. As tumours grew to 80 mm^3^, we divided the nude mice into four groups stratified according to volume: PBS control group and different doses of DCH intervention groups (25, 50, and 100 mg/kg). The tumour diameter was measured once every two days by a Vernier calliper. The tumour volume was calculated (volume calculation formula: (length in mm) (width in mm)^2^/2). The mice were sacrificed after two weeks, and the serum was collected for liver and kidney function assessments and detection of myocardial markers. Tumours were simultaneously collected and weighed. Some of the tumour specimens were fixed in 4% paraformaldehyde, and other specimens were frozen in liquid nitrogen.

### 2.7. Western Blot

Protein was extracted from tumour tissues of nude mice and G401 cells treated with DCH and lysed using RIPA buffer containing 1% protease inhibitor, and the protein concentration was determined by BCA assay. An exact amount of protein (20 *μ*g) was separated by sodium dodecyl sulfate–polyacrylamide gel electrophoresis (SDS–PAGE) and transferred to polyvinylidene fluoride (PVDF) membranes (Microwell, USA). The membranes were then blocked with 5% skim milk for 1 h and incubated with primary antibody overnight at 4°C. The primary antibodies were as follows: MMP2 (1 : 1000, Affinity Biosciences, Cat No. #AF5330), MMP9 (1 : 1000, Affinity Biosciences, Cat No. #AF5228), E-cadherin (1 : 1000, ZENBIO, Cat No. 201283), ZO-1 (1 : 1000, Affinity Biosciences, Cat No. #AF5145), *α*-SMA (1 : 1000, ZENBIO, Cat No. 380653), N-cadherin (1 : 1000, Proteintech, Cat No. 66219-1-Ig), vimentin (1 : 1000, ZENBIO, Cat No. R22775), and GAPDH (1 : 5000, ZENBIO, Cat No. 200306-7E4). The next day, the samples were washed 3 times using TBST, incubated with secondary antibodies at room temperature for 1 h, washed 3 times using TBST, and imaged using chemiluminescence, employing statistical grey values from Image Lab.

### 2.8. Immunofluorescence

Tumour tissue specimens from MRTK were fixed in 4% paraformaldehyde, embedded in paraffin, cut into 4 *μ*m thick sections, routinely dewaxed in water, and blocked with 0.5% bovine serum albumin (BSA) for 1 hour after antigen repair. The primary antibody was incubated overnight at 4°C, washed three times in PBS, incubated with the corresponding fluorescent secondary antibody for 1 h, washed 3 times in PBS, stained with DAPI for 30 min, and sealed with an antifluorescent quencher. Finally, photographs were taken using a fluorescence microscope (Olympus Crops, Tokyo, Japan).

G401 cells were seeded into 24-well plates of prelaid cell crawl sheets. After DCH treatment, they were washed 3 times in PBS, fixed in 4% paraformaldehyde for half an hour, permeabilized with 0.2% Triton X-100, blocked in 0.5% BSA for 1 hour, and incubated with primary antibody at 4°C overnight. The remaining steps were consistent with the immunofluorescence assay for tissue samples.

The primary antibodies used for immunofluorescence included MMP2, MMP9, E-cadherin, N-cadherin, ZO-1, *α*-SMA, vimentin, and *β*-tubulin antibodies, which were diluted in 0.5% BSA at a ratio of 1 : 200.

### 2.9. Cytoskeleton Detection

For F-actin staining, we stained cells with rhodamine-labelled phalloidin. The cells were routinely fixed with 4% polyformaldehyde, blocked with 0.5% BSA, stained with phalloidin for 1 h, washed 3 times with PBS, stained with DAPI for 30 min, washed 3 times with PBS, and sealed with an antifluorescence quencher. The *β*-tubulin staining method was the same as that described for the cellular immunofluorescence assay, and the cells were photographed under a confocal microscope.

### 2.10. Data Statistics

All data are expressed as the mean ± standard deviation (SD), and statistical analysis was performed using GraphPad Prism software (GraphPad 6.0). Student's *t*-test was used to detect statistically significant differences between two groups, and one-way ANOVA was used to compare data from more than three groups. *P* < 0.05 was considered statistically significant, and all experiments were repeated at least three times independently.

## 3. Result

### 3.1. Differential Expression of MMP2 and MMP9 in MRTK

**Figure 1:**
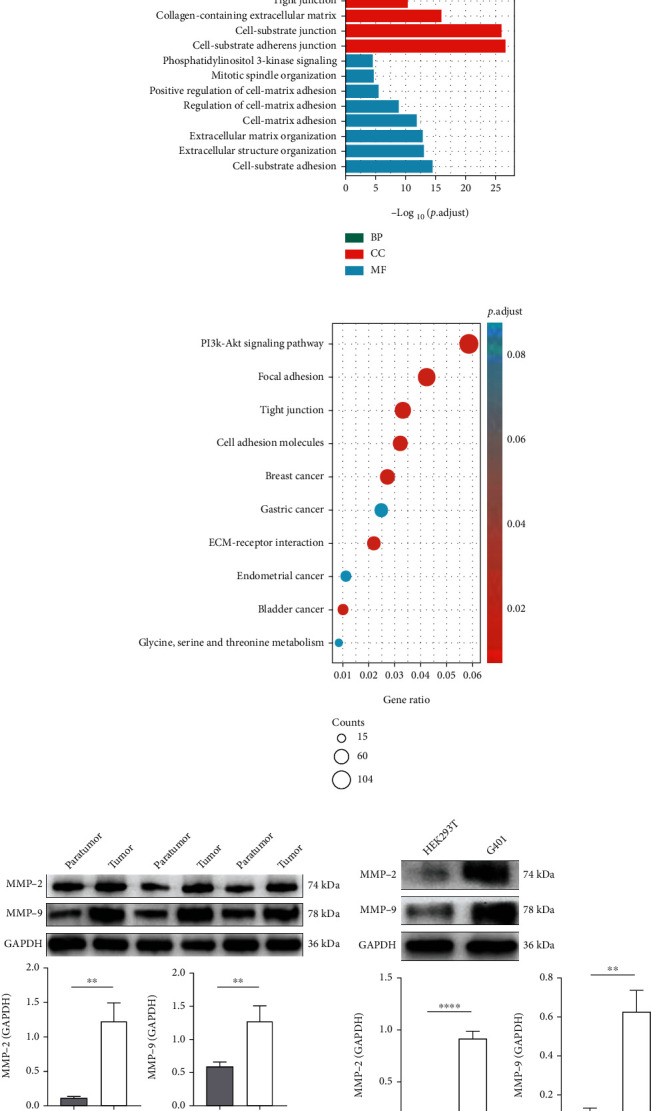
Differential expression of MMP2 and MMP9 in MRTK. (a) GO analysis of the differentially-expressed genes in MRTK. (b) KEGG analysis of the differentially-expressed genes in MRTK. (c) The expression of MMP2 and MMP9 in clinical samples of MRTK (*n* = 6). (d) The expression of MMP2 and MMP9 in G401 cells. ^∗∗^*p* < 0.01; ^∗∗∗∗^*p* < 0.0001.

We downloaded MRTK sequencing data from the TARGET database. GO analysis (Figure 1(a)) and KEGG analysis (Figure 1(b)) of the differentially-expressed genes revealed multiple enrichments in ECM-related functions and pathways, such as ECM binding and cell matrix adhesion. MMP2 and MMP9 are critical components for ECM degradation. Therefore, we further examined the expression levels of MMP2 and MMP9 in clinical samples and the G401 cell line of MRTK. The results showed that MMP2 and MMP9 were significantly highly expressed in MRTK tumour tissues compared to adjacent cancerous tissues, and MMP2 and MMP9 were equally highly expressed in G401 cells compared to control HEK293T cells (Figure 1(d)).

### 3.2. EMT Is Present in MRTK

**Figure 2:**
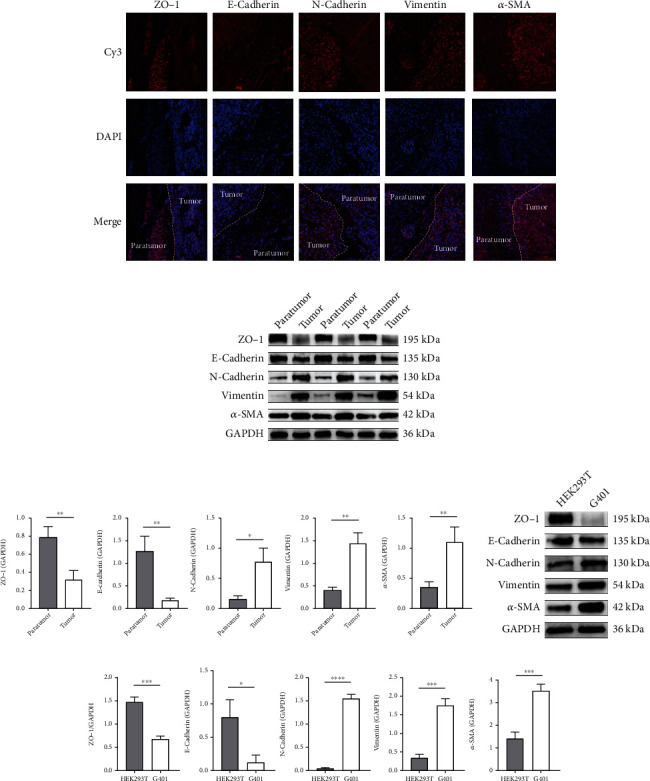
The EMT phenomenon exists in MRTK. (a) Immunofluorescence of EMT-related indicators in MRTK clinical samples. (b) WB of EMT-related indicators in MRTK clinical samples. (c) Statistical map of related indicators in Figure 2(b). (d) WB of EMT-related indicators in G401 cells. (e) Statistical map of related indicators in Figure 2(d). ^∗^*p* < 0.05;  ^∗∗^*p* < 0.01;  ^∗∗∗^*p* < 0.001;  ^∗∗∗∗^*p* < 0.0001.

EMT is considered necessary for tumourigenesis. To verify the presence of EMT in MRTK cells, we examined the expression profiles of ZO-1 and E-cadherin, which are epithelial-related markers, and the mesenchymal markers N-cadherin, vimentin, and *α*-SMA using immunofluorescence and WB in clinical samples. The results showed that ZO-1 and E-cadherin were significantly downregulated in tumour tissue, while N-cadherin, vimentin, and *α*-SMA were upregulated (Figures 2(a)–2(c)). We further examined the expression of the above indexes in G401 cells and control HEK293T cells using WB, and the results showed that the epithelial-related indicators ZO-1 and E-cadherin were significantly downregulated. In contrast, the mesenchymal-related indicators N-cadherin, vimentin, and *α*-SMA were significantly upregulated in G401 cells (Figures 2(d) and 2(e)).

### 3.3. DCH Inhibits the Expression of MMP2 and MMP9 and the Proliferation, Migration, and Invasion of G401 Cells

**Figure 3:**
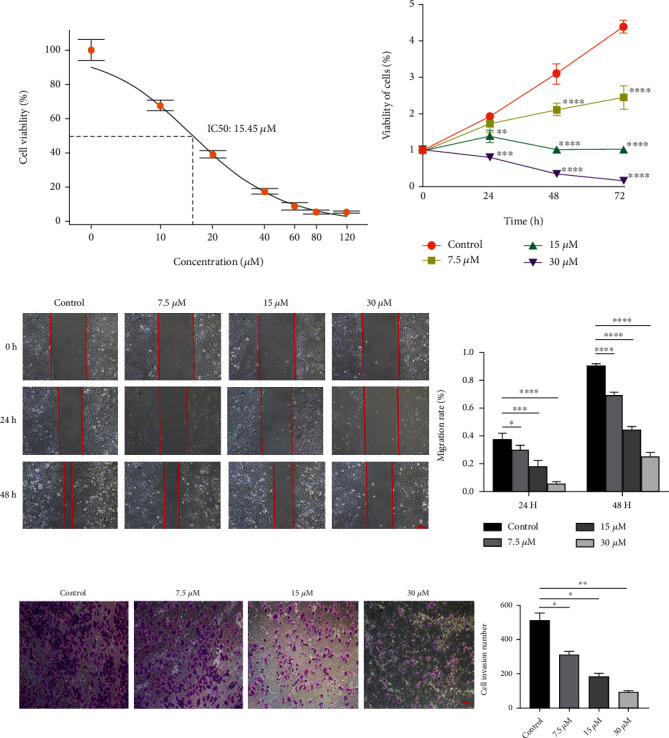
DCH inhibits the proliferation, migration, and invasion of G401 cells. (a) Viability detection of G401 cells with different concentrations of DCH intervention. (b) CCK8 detects the effect of DCH on cell proliferation. (c) Scratches detect the effect of DCH on the migration ability of G401 cells. (d) Transwell assays detect the effect of DCH on the invasive capacity of G401 cells. ^∗^*p* < 0.05;  ^∗∗^*p* < 0.01;  ^∗∗∗^*p* < 0.001;  ^∗∗∗∗^*p* < 0.0001.

**Figure 4:**
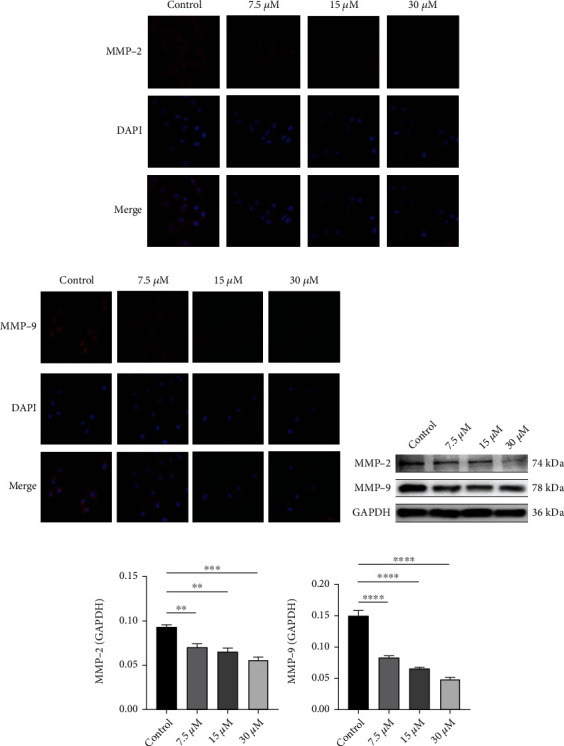
DCH inhibited the expression of MMP2 and MMP9 in G401 cells. (a) Fluorescence expression of MMP2 in G401 cells after doxycycline intervention. (b) MMP9 fluorescence expression in G401 cells after doxycycline intervention. (c) WB expression of MMP2 and MMP9. (d) Statistical map of MMP2 and MMP9 expression in WB experiments. ^∗^*p* < 0.05;  ^∗∗^*p* < 0.01;  ^∗∗∗^*p* < 0.001;  ^∗∗∗∗^*p* < 0.0001.

We explored the effect of DCH on the proliferation, migration, and invasion capacity of G401 cells. We treated G401 cells with different concentrations of DCH and determined the concentration of the subsequent intervention, which showed an IC_50_ of 15.45 *μ*M, close to 15.0 *μ*M (Figure 3(a)). Therefore, we selected 7.5, 15, and 30 *μ*M DCH for subsequent cell experiments. The results of the CCK-8 proliferation experiment showed that DCH significantly inhibited G401 cell proliferation in a dose-dependent manner (Figure 3(b)). The scratch experiment showed that DCH significantly inhibited G401 cell migration in a dose-dependent manner (Figure 3(c)). The Transwell results showed that DCH significantly inhibited the invasive capacity of G401 cells in a dose-dependent manner (Figure 3(d)). Based on confirming the presence of high MMP2 and MMP9 expression in MRTK, we further examined the expression of MMP2 and MMP9 in G401 after DCH intervention. Immunofluorescence and WB results showed that MMP2 and MMP9 expression levels were indeed reduced in G401 cells after DCH intervention (Figures 4(a)–4(d)).

### 3.4. DCH Can Regulate Cytoskeletal Rearrangement and Inhibit EMT in G401 Cells

**Figure 5:**
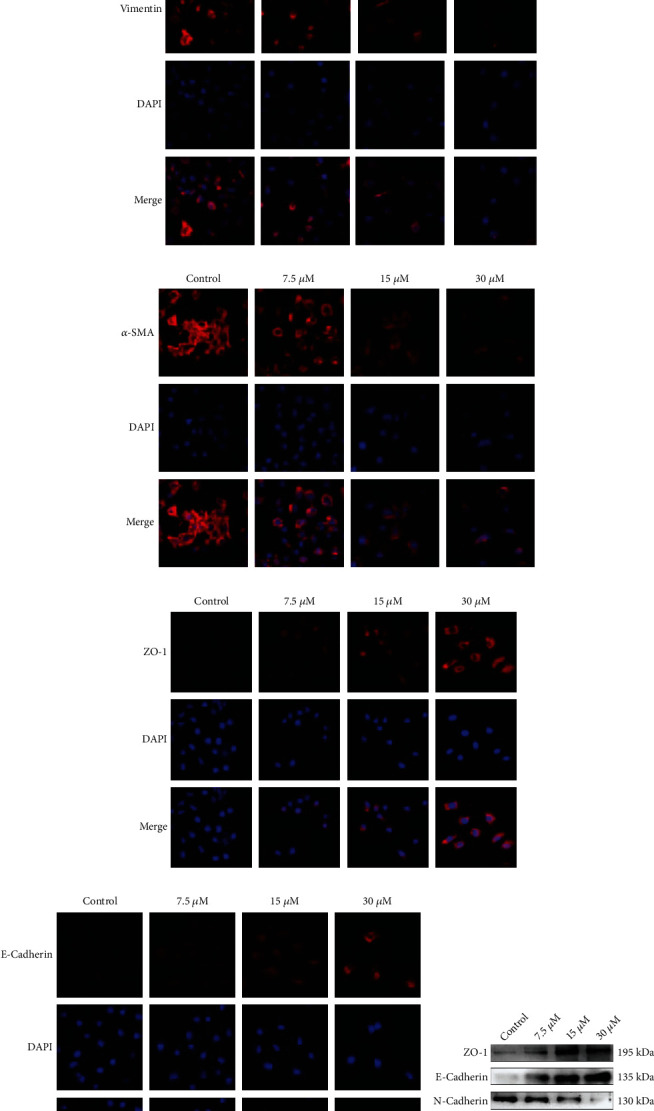
DCH inhibited EMT in G401 cells. (a–e) Changes in the immunofluorescence expression of EMT-related indicators in G401 cells after DCH intervention. (f) WB diagram of EMT-related indicators in G401 cells after DCH intervention. (g) Statistical map of related indicators in (f). ^∗^*p* < 0.05;  ^∗∗^*p* < 0.01;  ^∗∗∗^*p* < 0.001;  ^∗∗∗∗^*p* < 0.0001.

**Figure 6:**
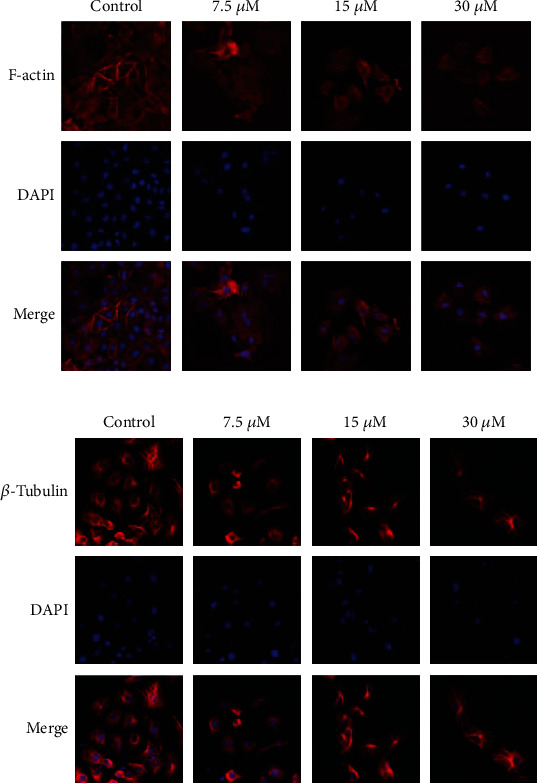
G401 cytoskeleton changes after DCH intervention. (a) F-actin changes in G401 cells after DCH intervention. (b) *β*-Tubulin change in G401 cells after DCH intervention.

To verify whether DCH could reverse the EMT phenomenon of MRTK, we further examined the changes in epithelial-related markers and mesenchymal markers in G401 cells in each group after DCH intervention. Immunofluorescence showed that G401 N-cadherin, vimentin, and *α*-SMA expression levels were significantly reduced, while the expression levels of the epithelial-related indexes ZO-1 and E-cadherin were significantly enhanced (Figures 5(a)–5(e)). The WB results showed the same trends (Figures 5(f) and 5(g)). The above results indicated that DCH inhibited EMT in G401 cells. Because EMT reflects the remodelling of the cytoskeleton, we further examined the changes in cytoskeletal F-actin and *β*-tubulin after DCH intervention. The results showed a rearrangement of cytoskeletal F-actin and *β*-tubulin after DCH intervention. F-actin staining showed that the microfilaments became shorter and became arranged more neatly than before medication, and the cell morphology became round. Microtubules of control cells from the perinucleus to the surrounding cytoplasm showed a more uniform network structure; the cell microtubule arrangement changed significantly after DCH intervention, the microtubule skeleton reorganization was more significant in the high-dose DCH intervention group, and perinuclear microtubules were emitted in a radial arrangement from centrioles (Figures 6(a) and 6(b)). The above results suggest that DCH may inhibit EMT through the regulation of G401 cytoskeletal rearrangement.

### 3.5. DCH Inhibited Tumour Growth in Tumour-Bearing Mice and Showed No Significant Damage to Heart, Liver, or Kidney Function

**Figure 7:**
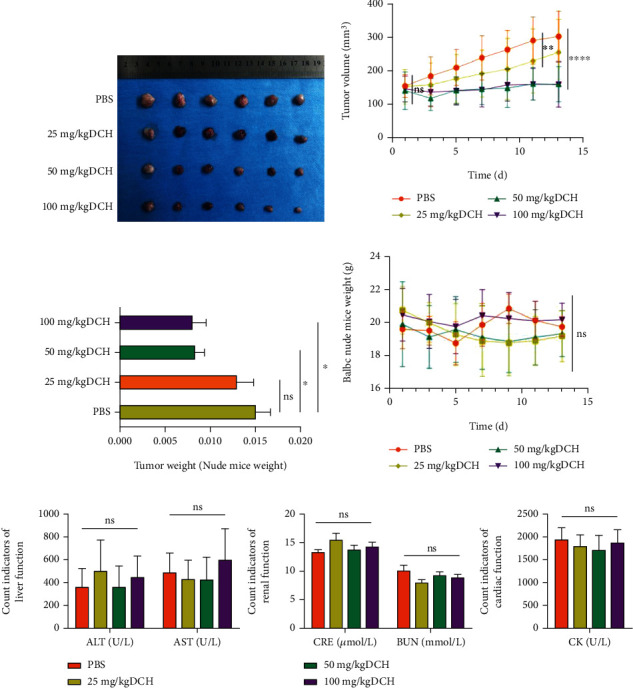
Effect of DCH on tumour growth and vital organ function in tumour-bearing mice. (a) General tumour chart of each group after DCH intervention. (b) Trend of tumour volume in each group. (c) Tumour weight/mouse weight in each group. (d) Weight trend of mice. (e) Liver and kidney function and myocardial markers in each group after DCH intervention. ^∗^*p* < 0.05;  ^∗∗^*p* < 0.01;  ^∗∗∗^*p* < 0.001;  ^∗∗∗∗^*p* < 0.0001.

We further examined the effects of DCH on the growth of tumour tissues in G401 tumour-bearing mice by *in vivo* experiments. We found that the tumour volume decreased significantly with DCH compared with the PBS control group. The medium and high concentrations significantly inhibited tumour growth compared with the low-concentration group, with no significant difference between the middle- and high-concentration groups (Figures 7(a) and 7(b)). However, the tumour volume to weight ratio showed no significant difference in volume/weight in the low-concentration DCH group compared with the PBS control group, while there were significant decreases in the middle- and high-concentration groups, and there was no significant difference between the two groups (Figure 7(c)). The mouse growth curve showed no significant difference in body weight in each group (Figure 7(d)). The safety of DCH *in vivo* was further evaluated. The expression of liver and kidney and myocardial markers after PBS and DCH intervention was examined, and the results showed no significant differences (Figure 7(e)) in ALT, AST, CRE, BUN, and CK in the DCH group compared with the PBS group. The above results showed that DCH could inhibit tumour growth *in vivo* and that DCH did not show significant damage to liver and kidney function or myocardial markers in mice.

### 3.6. DCH Inhibits MMP2 and MMP9 Expression in Mouse Tumour Tissues

**Figure 8:**
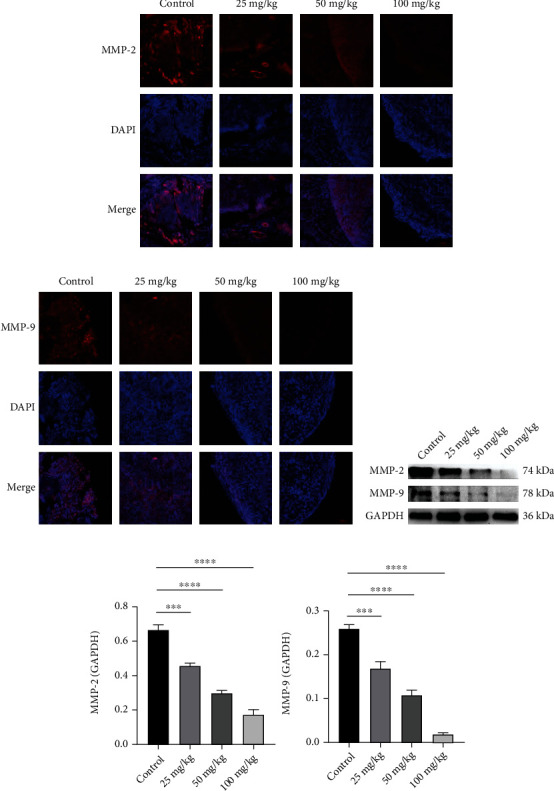
Changes in MMP2 and MMP9 in tumour tissues of tumour-bearing mice after DCH intervention. (a) MMP2 fluorescence expression of tumour tissue of mice after DCH intervention. (b) MMP9 fluorescence expression of tumour tissue of mice after DCH intervention. (c) WB diagram of MMP2 and MMP9 of tumour tissue of each group after DCH intervention. (d) Statistical map of MMP2 and MMP9. ^∗^*p* < 0.05;  ^∗∗^*p* < 0.01;  ^∗∗∗^*p* < 0.001;  ^∗∗∗∗^*p* < 0.0001.

We further examined MMP2 and MMP9 expression in the tumours of mice after DCH intervention by immunofluorescence and WB and showed that MMP2 and MMP9 expression levels were significantly reduced in the tumour tissue of DCH-infused mice, and the decreases were dose-dependent (Figures 8(a)–8(d)).

### 3.7. DCH Inhibited the EMT of MRTK *In Vivo*

**Figure 9:**
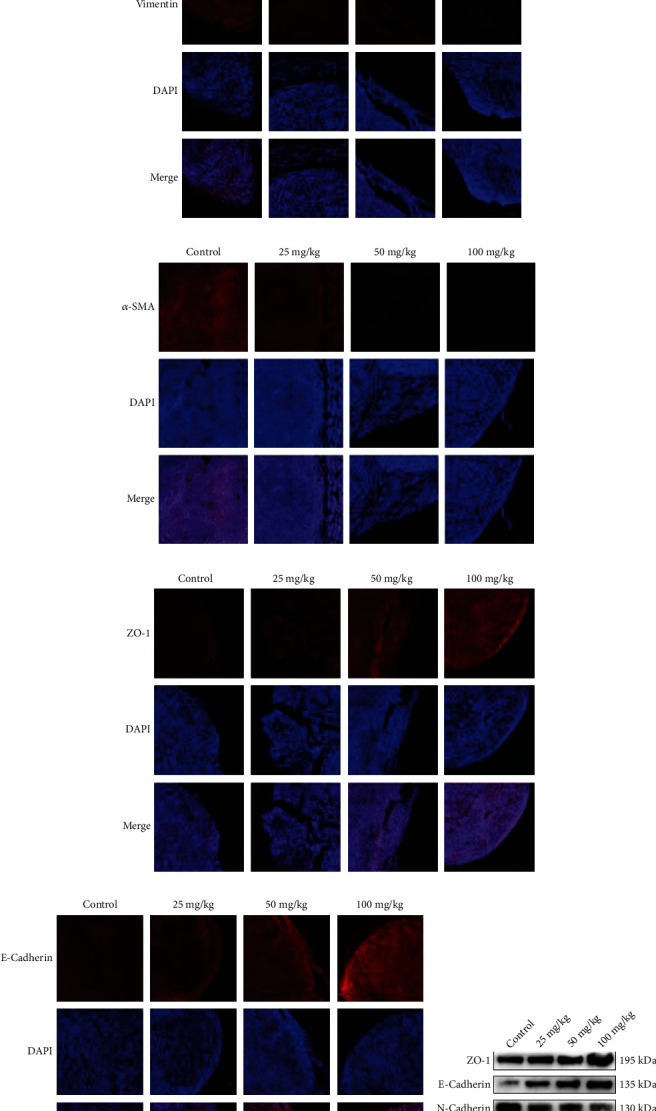
EMT of mouse tumour tissue after DCH inhibition. (a–e) Changes in the immunofluorescence expression of EMT-related indexes in the tumour tissue of mice after DCH intervention. (f) WB diagram of EMT-related indexes in the tumour tissue of mice after DCH intervention. (g) Statistical map of related indexes in (f). ^∗^*p* < 0.05;  ^∗∗^*p* < 0.01;  ^∗∗∗^*p* < 0.001;  ^∗∗∗∗^*p* < 0.0001.

We further tested whether DCH could inhibit the EMT of MRTK *in vivo*. The immunofluorescence results showed that the expression levels of N-cadherin, vimentin, and *α*-SMA were significantly decreased, while the levels of ZO-1 and E-cadherin were significantly enhanced (Figures 9(a)–9(e)). The expression was further detected using WB and showed the same trends (Figures 9(f) and 9(g)). The above results indicated that DCH inhibited the EMT of MRTK *in vivo*.

## 4. Discussion

Currently, tumours have become the second leading cause of death in children, and an increasing amount of research is committed to finding new treatment options. MRTK is an embryonal tumour occurring in the kidney and is extremely rare due to its low incidence; therefore, both basic and clinical studies on MRTK are rare. However, due to its highly aggressive nature and rapid progression characteristics, the prognosis of children with MRTK is poor. The migration and invasion of tumour cells require proteolytic enzymes, which degrade the ECM and allow tumour spread. MMPs are most closely associated with metastatic spread [35, 36]. For example, MMP1 overexpression can promote EMT through ECM degradation, leading to the enhanced invasion and migration of hepatocellular carcinoma (HCC) cells [37]. Given considerable evidence linking MMPs with tumour progression and poor prognosis, researchers synthesized and tested several MMPIs, including batimastat, marimastat, and tanomastat. However, broad-spectrum or selective MMP inhibitors showed sound antitumour effects in pancreatic and breast cancer due to various toxic side effects and low bioavailability [38].

DCH is a broad-spectrum MMP inhibitor that has been widely used in clinical studies as a therapy for a variety of malignancies. Studies have shown that DCH suppresses the growth of diffuse large B-cell lymphoma [39], and positive effects were reported in recent studies of lymphoma patients [40]. Moreover, current studies have shown that DCH can reduce the activity levels of MMPs, particularly MMP2 and MMP9, in breast cancer, glioma, and oral squamous cell carcinoma [28, 41, 42]. There is evidence that MMPs can act as promoters and mediators to induce EMT or EMT-related processes in cultured cells, potentially interfering with MMP-mediated EMT by targeting MMPs for catalytic inhibition [43]. Meng et al. confirmed that DCH inhibited EMT progression and vimentin formation in HCC by inhibiting MMP activity [44]; some studies showed that DCH could inhibit stem cell properties and EMT in pancreatic cancer and breast cancer and progression [25, 45]. The above studies suggest that DCH, acting as an MMP inhibitor, may inhibit tumour EMT and tumour progression by regulating MMP activity.

MRTK, as a highly malignant tumour, is highly susceptible to metastasis and invasion. Our study is aimed at determining whether DCH has an antitumour effect on MRTK and the relevant mechanisms of its antitumour effect. Our KEGG and GO analyses using data from the TARGET database found that pathways such as ECM were mainly enriched, and MMP2 and MMP9 are fundamental for ECM degradation; therefore, we speculated that MMP2 and MMP9 play vital roles in the progression of MRTK. We validated this possibility in G401 cells and the tumour tissues of MRTK and confirmed the high expression levels of MMP2 and MMP9 in MRTK. Because component changes in ECM induce EMT, MMP2 and MMP9 can also induce and promote EMT to promote tumour progression. Therefore, we further verified EMT in MRTK cells. EMT includes loss of epithelial cell markers such as decreased expression levels of E-cadherin and zona occludens 1 (ZO-1) [46]; E-cadherin is a tumour suppressor protein with loss of expression associated with EMT during tumour metastasis [47]. ZO-1 is a membrane scaffold protein that plays a vital role in maintaining tight junction integrity in many invasive cancers [48]. The EMT process also includes the upregulation of mesenchymal markers, such as vimentin, *α*-SMA, and N-cadherin. Vimentin is expressed in various epithelial carcinomas. Moreover, its overexpression is closely associated with accelerated tumour growth, invasion, and poor prognosis [49]; *α*-SMA was confirmed to be highly expressed in breast cancer [50]; aberrant expression of N-cadherin has been found in many cancers, and its abnormal expression is closely related to the transformation, adhesion, apoptosis, angiogenesis, invasion, and metastasis of human malignancies [51]. Studies have also shown that *α*-SMA is a biomarker of malignant transformation in ameloblastoma [52]. Our results showed that in tumour tissues and G401 cells, the expression levels of the epithelial markers E-cadherin and ZO-1 were decreased, while the expression levels of the mesenchymal markers vimentin, *α*-SMA, and N-cadherin were increased, indicating EMT in MRTK. Therefore, we speculate that the high expression levels of MMP2 and MMP9 promote EMT in MRTK, thus promoting tumour migration and invasion and leading to malignant tumour progression.

We further explored whether DCH has an antitumour effect on MRTK and whether DCH exerts an antitumour effect by reversing EMT and inhibiting the expression of MMP2 and MMP9. We first examined the expression of MMP2 and MMP9 in G401 cells and mouse tumour-bearing tissues after intervention with DCH. We found that MMP2 and MMP9 were significantly reduced after DCH administration, indicating that DCH may exert subsequent antitumour effects by inhibiting MMP2 and MMP9. Our cellular experiments showed that DCH inhibited the proliferation, migration, and invasion of G401 cells *in vitro* in a dose-dependent manner. *In vivo*, DCH also significantly inhibits tumour growth and causes no apparent liver, kidney, or myocardial marker damage, showing some safety. We further examined the changes in EMT-related indicators, which showed that epithelial-related markers such as E-cadherin and ZO-1 were increased and mesenchymal markers such as vimentin, *α*-SMA, and N-cadherin were decreased after doxycycline treatment, indicating that doxycycline can inhibit EMT in MRTK.

To exit the primary tumour and invade the surrounding tissues, tumour cells must disrupt cell–cell contact. During EMT, the epithelial cytoskeleton is reconstructed, leading to loss of cell polarity, destruction of cell–cell junctions, degradation of the underlying basement membrane, and reorganization of the ECM. Then, the cells begin to invade [14]. At the same time, studies show that the nature of EMT is the remodelling of the cytoskeleton. The actin cytoskeleton is a highly dynamic structure that is constantly reconstructed in living cells. This regulation is a prerequisite for processes such as cell motility and cancer cell invasion [53]. The cytoskeleton is mainly composed of microtubules and microfilaments, and it has been reported that the stabilization of F-actin can maintain cell–cell junctions (adhesion and tight junctions). In early EMT, F-actin changes dynamically, which weakens cell–cell adhesion and causes cell connection instability [54]. Microtubules are an essential part of the cytoskeleton and play important roles in cell movement, intracellular transport, and cell shape support [55]. Therefore, we further examined the expression of F-actin and tubulin in G401 cells after DCH intervention and showed significant cytoskeleton rearrangement, shorter microfilaments, a more orderly arrangement than before treatment, and blunted cell morphology after DCH intervention. Microtubules undergo significant reorganization, changing from a uniform network emitted around the nucleus to a radial arrangement originating from the perinuclear periphery, with less mesh structure. It was confirmed that DCH induced cytoskeletal rearrangement and thus reversed EMT.

## 5. Conclusions

Taken together, our study is the first to demonstrate the high expression levels of MMP2 and MMP9 in MRTK and the presence of EMT in MRTK. In addition, DCH, as an MMP inhibitor, can inhibit the activity of MMP2 and MMP9 in MRTK and regulate cytoskeletal rearrangement, causing partial reversal of EMT. This suggests that the antitumour effect of doxycycline on MRTK may be mediated by reversing EMT by regulating MMP2 and MMP9 activity as well as by cytoskeletal rearrangements. A number of studies have focused on the antitumour effects of DCH, and these studies are also highly consistent with our conclusions. However, there are also reports that DCH can promote tumour progression. For example, Zhu et al. confirmed that DCH could regulate gene expression to induce liver tumours [56]. Therefore, there are two opposite views of the effect of DCH on tumours. Based on the failure of previous MMP inhibitors in clinical research due to various side effects and low validity, we believe that if DCH can be successfully applied in the clinic, we need to strive to avoid toxic side effects and improve its biological efficacy. The current research hotspot is to use nanocarriers to wrap small molecule chemotherapy drugs, thus reducing their toxic side effects. At present, some researchers have successfully prepared nanoparticles (DOXY-PNPs) coated with DCH and showed great antitumour potential for solid Ehrlich carcinoma (SEC) [57], showing the application prospect of nanomaterials wrapped with DCH. The results of this study demonstrate the prospect of DCH in MRTK; therefore, we plan to prepare oral drugs with milk exosomes containing DCH in our subsequent study to further verify its antitumour effect *in vivo*.

## Data Availability

The TARGET data analyzed in this study is available at https://ocg.cancer.gov/programs/target.
